# Selection of patients with left breast cancer for IMRT with deep inspiration breath-hold technique

**DOI:** 10.1093/jrr/rraa003

**Published:** 2020-03-03

**Authors:** Chih-Shen CHANG, Chia-Hsin CHEN, Kuo-Chi LIU, Chia-Sheng HO, Miao-Fen CHEN

**Affiliations:** 1 Department of Radiation Oncology, Chang Gung Memorial Hospital at Chia-Yi, Putz City, Taiwan; 2 College of Medicine, Chang Gung University College of Medicine, Taiwan

**Keywords:** breast cancer, DIBH, heart, IMRT, lung volume

## Abstract

The deep inspiration breath-hold (DIBH) technique has been utilized to reduce the cardiac dose in left-sided breast cancer (BC) patients undergoing radiotherapy. Further investigation of the parameters for selecting which patients will benefit most from DIBH is essential. We performed dosimetric comparisons for 21 patients with left-sided BC who had both computed tomography (CT)-based free-breathing (FB) and DIBH plans. The doses to the heart and left anterior descending artery (LAD) and any reduction due to the DIBH technique were analysed. Based on CTFB plans, dosimetric analysis revealed that the irradiation doses to the heart and LAD were significantly correlated with the target volume, the ipsilateral lung volume (ILV) and the total lung volume (TLV). When patients had an ILV ≥ 950 cm^3^ or a TLV ≥ 2200 cm^3^, the irradiation doses to the heart and LAD were significantly decreased. Furthermore, the reduction in the mean heart dose (MHD) was correlated to the difference in lung volume between FB and DIBH. The difference in ILV between DIBH and FB of 1.8 indicated that the patients obtained more benefit from the DIBH technique. The data suggest that lung volume (ILV and TLV) measured on a CT-simulation scan and the difference between FB and DIBH could be utilized to help select patients for DIBH.

## INTRODUCTION

Postoperative radiotherapy (RT) provides a substantial reduction in local and regional recurrence rates for breast cancers (BCs) and contributes to improvements in overall survival [1, 2]. Despite improved disease control, RT increases the risk for cardiovascular disease in BC patients [3, 4]. RT-induced heart disease is correlated with the absorbed dose and irradiated volume [5, 6]. The higher the volume of the heart included in the radiation field, or the higher the dose to the heart, the higher the likelihood of cardiac injury leading to ischemic heart disease and cardiac mortality [7]. Darby *et al*. [8] identified a linear relationship between the mean heart dose (MHD) and the rate of major coronary events, which increased by 7.4% per Gy of the MHD. Occlusion of the left anterior descending artery (LAD) has been reported to cause a large area of myocardial necrosis and to lead t o severe left ventricle impairment and congestive heart failure [9, 10]. Furthermore, the MHD has been significantly correlated with the mean dose (Dmean) to the LAD [9, 11]. This observation can be explained by the fact that the highest radiation doses are likely to be to the anterior portion of the heart, including the LAD, by tangential-field RT for BC. The use of intensity-modulated RT (IMRT) has been shown to reduce the heart dose [12–14]. Therefore, this study analysed the organs at risk (OAR) dose metrics for left-sided BC patients treated with IMRT.

Deep inspiration breath-hold (DIBH) with gating has been reported as an option to reduce exposure to the heart and coronary arteries [15–18]. It has been indicated that left-sided BC patients should benefit from the DIBH technique, but not all patients have the same benefits, and the relative reduction of MHD has ranged from 26.2% to 75% in previous studies [19, 20]. Investigation into the parameters for selecting patients who are more likely to benefit from DIBH is essential. Previously, patients were selected based on age, BMI, and the ability to breath-hold for comparisons of the respective dose–volume histograms (DVHs) [15, 21, 22]. A devised selection criterion has also remained in operation. Accordingly, in the present study, we further analysed individual dosimetric reduction through the use of the DIBH technique to select which patients would benefit the most.

## MATERIALS AND METHODS

### Characteristics of patients and treatment technique

This retrospective study was approved by the hospital review board. The patients included women who had undergone breast-conserving surgery (BCS) (18 patients) or mastectomy (three patients) for left-sided BC, followed by adjuvant RT with IMRT, in our department between February and June 2019. In general, adjuvant IMRT was prescribed according to the planned target volume (PTV), which was 50 Gy in 25 fractions (nine patients) or 42.56 Gy in 16 fractions (12 patients). Of the 21 patients, 9 received simultaneous nodal irradiation. For patients with intact breast tissue following BCS, the clinical target volume (CTV) was defined as the whole breast. For postmastectomy patients, the CTV encompassed from 5 mm below the skin surface and included the soft tissues down to the deep fascia. For patients who required simultaneous nodal irradiation, the supraclavicular lymph nodes were included in the CTV. The CTV was expanded by 10 mm, but within 2 mm of the skin surface, to create the PTV. RT was planned using the Eclipse planning system, and treatment was delivered using the Varian Edge radiosurgery system. The goal was to deliver 95% of the prescribed dose to at least 95% of the PTV while minimizing the dose delivered to the lungs, heart, and contralateral breast. The treated volume was defined as the volume enclosed within the prescribed dose. Relevant dose–volume data were retrieved from DVH.

### Organs at risk

The heart, LAD, and ipsilateral and contralateral lungs were defined as organs at risk (OARs). The dose constraint (in 2 Gy per fraction) of the heart was V25 Gy < 10% and MHD < 4 Gy, and the criteria for the whole lung were V20 < 20% and V5 Gy < 30%. For the patients treated with 42.56 Gy in 16 fractions (hypofractionation regimen), the dose to the heart and LAD were corrected to 2 Gy per fraction, a/b = 2.5 Gy, and the dose for the lung was corrected to 2 Gy per fraction, a/b = 3 Gy [23]. For lung volume in FB, the mean left ipsilateral lung volume (ILV) and total lung volume (TLV) of all 21 patients were 973 ± 35.6 cm^3^ and 2276 ± 89.6 cm^3^, respectively. To assess the predictive value of the ILV and TLV, they were each redefined as a binary variable by finding the value from the receiver-operating characteristic (ROC) curve that maximized the percentage correctly classified for D2cm^3^ < 42 Gy. The optimal cut-offs for ILV and TLV were 952 and 2199, respectively. Accordingly, we divided the patients into two ILV groups (around 950 cm^3^) and two TLV groups (around 2200 cm^3^) for further analysis.

### CT-simulation

At the time of simulation, these patients were instructed in the DIBH technique and respiratory gating devices, including the Real-Time Position Management System (RPM; Varian Medical Systems, Palo Alto, CA, USA) and a visual coaching device (VCD). Patients underwent two CT scans, one with FB and one with DIBH, and the corresponding computed tomography (CT) plans for FB and DIBH were termed the CTFB and CTDIBH plans. All patients underwent left-breast RT treatment with the DIBH technique in the study period.

### Statistical analysis

Student’s *t*-test was used to compare the dose distribution between the plans. The *P*-value for a two-tailed test with a confidence interval of 95% was used. Linear regression analysis was performed using SPSS version 17.0. Dose reduction to OARs was compared between the FB and DIBH plans.

## RESULTS

### Dosimetric evaluation of CT-simulation scans based on free-breathing

#### OAR doses

**Fig. 1.**
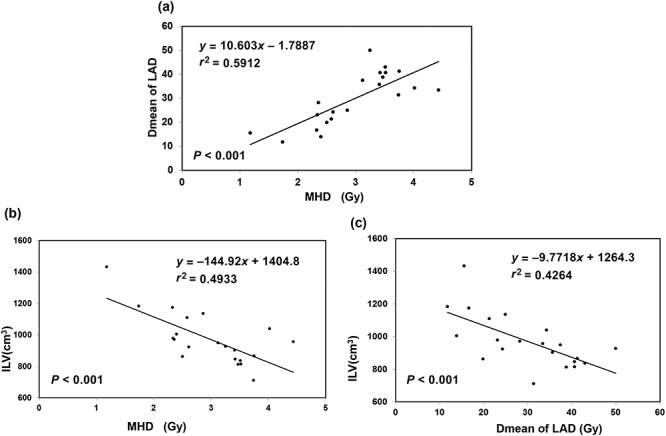
Relationship between MHD, Dmean of LAD and ILV in CTFB plans for left-sided breast cancer patients. MHD = mean heart dose, Dmean = mean dose, LAD = left anterior descending artery, ILV = ipsilateral lung volume, CTFB = computer tomography–based free-breathing.

**Fig. 2.**
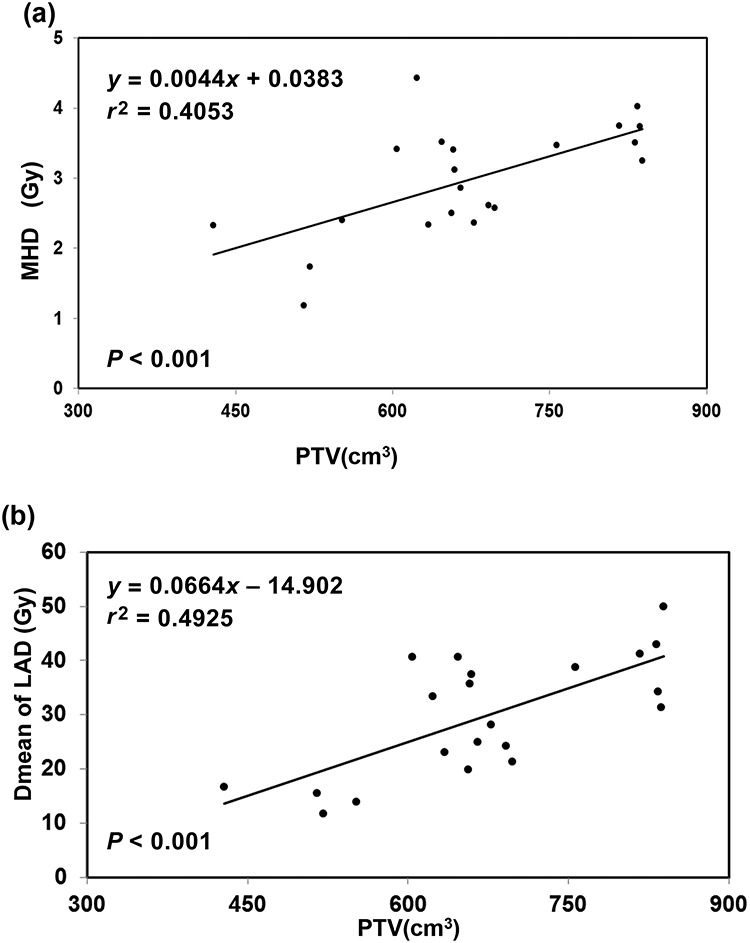
Relationship between MHD, Dmean of LAD and PTV in CTFB plans for left-sided breast cancer patients. MHD = mean heart dose, Dmean = mean dose, LAD = left anterior descending artery, PTV = planning target volume, CTFB = computer tomography–based free-breathing.

Each patient had CTFB and CTDIBH plans. These patients were analysed based on the DVHs for the CTFB plans. In our study, patient variability was noted in terms of the MHD, with a range of values from 1.18 to 4.43 Gy and a near-maximum dose of 2 cm^3^ to the heart (D2) from 12.2 to 49.44 Gy in patients with left-sided BC treated with IMRT without breathing control. In terms of other cardiac structures, the LAD was the most exposed, with mean doses ranging from 11.78 to 49.99 Gy. Linear regression analysis, shown in Fig. 1a, demonstrated a strong correlation with the MHD for the LAD through (*r* = 0.77), and the mean ratio of Dmean LAD/MHD was ~10. We also analysed the dose to the heart, and LAD correlated with the ILV and TLV. The analysis (Fig. 1b and c and Suppl. Fig. 1) revealed that the mean doses to the heart and the LAD were negatively correlated with the ILV and the TLV. Correlation analysis revealed that a larger ILV and TLV were associated with a lower MHD, D2, V25, V20 and V5 of the heart and a lower Dmean, V40 and V30 of the LAD. The median values of ILV and TLV were 949 cm^3^ and 2189 cm^3^, respectively. We further divided the patients into two ILV groups (above and below 950 cm^3^) and two TLV groups (above and below 2200 cm^3^). As shown in Table 1, ILV ≥ 950 cm^3^ (the high-ILV group) and TLV ≥ 2200 cm^3^ (the high-TLV group) were associated with lower doses to the heart and LAD. Supplementary Table 1 shows that patients with nodal irradiation had significantly greater heart doses (MHD and Dmean of LAD) and lung doses (Dmean, V5 and V20). There was no obvious difference in OAR dose parameters between patients who underwent BCS and those who underwent mastectomy. In terms of the target volume for patients with BCS, larger CTV and PTV in the CTFB plans were associated with a higher dose to the heart and LAD, including MHD, ΔD2cm^3^, V25, V20 and V5 to the heart, and a higher Dmean, V40 and V30 to the LAD. Regression analysis (Fig. 2a and b) revealed a positive correlation between the MHD and Dmean of the LAD with the PTV volume. However, the target volume had no significant effect on the dosimetric distribution to the ipsilateral or total lung (Dmean, V5 and V20).

**Table 1:** Difference in the irradiated dose of heart and LAD between the high and low groups of lung volume according to CTFB plans

Dosimetric parameter		TLV			ILV		
		Low	High	*P* value	Low	High	*P* value
Heart	**MHD (Gy)**	3.4 ± 0.14	2.4 ± 0.26	**0.003**	3.3 ± 0.12	2.6 ± 0.31	**0.047**
	**D2 (Gy)**	44.7 ± 1.35	33.3 ± 4.39	**0.012**	44.4 ± 1.43	34.8 ± 4.19	**0.037**
	**V25 (%)**	3.4 ± 0.6%	1.3 ± 0.3%	**0.016**	3.3 ± 0.7%	1.5 ± 0.4%	**0.043**
	**V20**	4.4 ± 0.7%	1.8 ± 0.4%	**0.010**	4.4 ± 0.7%	2.1 ± 0.5%	**0.024**
	**V5**	13.5 ± 1.2%	8.5 ± 1.2%	**0.010**	13.4 ± 1.3%	9.2 ± 1.3%	**0.035**
LAD	**Mean dose (Gy)**	36.3 ± 2.4	21.1 ± 2.4	**<0.001**	36.6 ± 2.6	22.3 ± 2.5	**0.001**
	**V30**	71 ± 7.7%	26 ± 7.5%	**0.001**	73 ± 8.2%	29 ± 7.3%	**0.001**
	**V40**	55 ± 7.1%	12 ± 5.4%	**<0.001**	56 ± 7.8%	16 ± 6.1%	**0.001**

### Dosimetric evaluation of the DIBH plan compared with the FB plan

All patients in the present study were treated with DIBH respiratory gating. They were examined based on the comparison of the respective DVHs for the CTFB and CTDIBH plans.

#### Planning volumes

The target coverage was comparable between the CTDIBH and CTFB plans. Table 2 shows the volumes of the CTV and the PTV and that of the prescribed dose. Both treatment plans were optimized using a similar strategy and yielded equivalent coverage of the PTV and CTV, represented by the relative volume covered by 95% of the prescribed dose (V95%).

**Table 2:** Dosimetric differences between CTFB and CTDIBH plans for target volume for all 21 patients

	FB	DIBH	*P* value
**CTV**			
Volume (cm^3^)^a^	427 ± 18	428 ± 18	0.96
V95(%)	99.5 ± 0.4%	99.6 ± 0.3%	0.88
**PTV**			
Volume (cm^3^)	673 ± 25	677 ± 24	0.91
V95(%)	94.7 ± 0.4%	95.3 ± 0.4%	0.70
**Treatment volume**			
Volume (cm^3^)	850 ± 42	877 ± 49	0.68
V105(%)	0.9 ± 0.2%	0.8 ± 0.2%	0.35

**Fig. 3.**
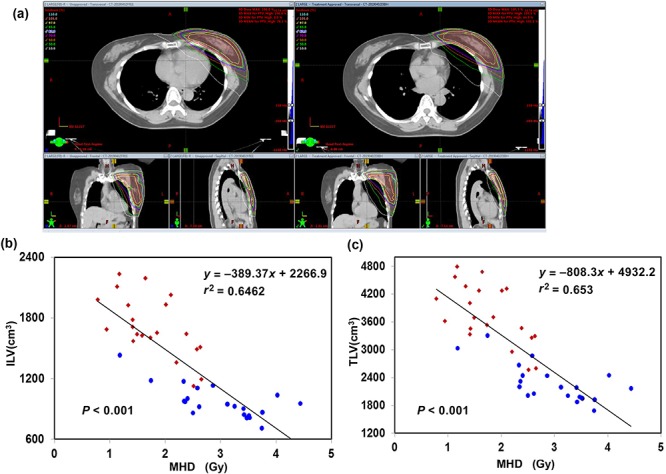
Dosimetric evaluation of the DIBH plan compared with the FB plan. (**a**) IMRT treatment plans with and without DIBH gating (dose: color curve; CTV: yellow curve; PTV: red colorwash). Left side: IMRT treatment planning with FB; right side: the same patient planned using gated breathing with DIBH. (**b**) Ipsilateral lung volume (ILV) and (**c**) total lung volume (TLV) plotted against mean heart dose according to the CTFB plan (blue) and the CTDIBH plan (red). ILV and TLV showed good negative linear correlations with mean heart dose for all patients. DIBH = deep inspiration breath-hold, FB = free-breathing, IMRT = intensity-modulated radiotherapy, CTV = clinical target volume, PTV = planning target volume, MHD = mean heart dose.

**Table 3:** Dosimetric differences from CTFB—CTDIBH plans for OAR for all 21 patients

	FB	DIBH	*P* value
**Heart**			
Dmean (Gy)^a^	2.97 ± 0.17	1.75 ± 0.12	<0.001
D2 (Gy)	39.8 ± 2.33	23.2 ± 2.35	<0.001
V3Gy (%)	19.1 ± 1.9%	11.4 ± 1.6%	0.003
V5Gy (%)	11.3 ± 1.0%	5.9 ± 0.8%	<0.001
V10Gy (%)	5.8 ± 0.7%	2.2 ± 0.4%	<0.001
V20Gy (%)	3.3 ± 0.5%	0.8 ± 0.2%	<0.001
V25Gy (%)	2.5 ± 0.5%	0.5 ± 0.1%	<0.001
V40Gy (%)	1 ± 0.2%	0.07 ± 0.03%	0.001
**LAD**			
Dmean (Gy)^a^	29.9 ± 2.8	17.4 ± 2.5	0.001
V30Gy (%)	51.8 ± 7.3%	21.2 ± 6.2%	0.003
V40Gy (%)	36.7 ± 6.6%	11.7 ± 5.2%	0.005
**Lung**			
Left Dmean (Gy)	10.2 ± 0.6	8.9 ± 0.5	0.108
Left V20Gy (%)	18.4 ± 1.4	15.7 ± 1.0	0.141
Left V5Gy (%)	39.8 ± 1.6	36.1 ± 1.5	0.101
ILV (cm^3^)^a^	973 ± 35	1716 ± 65	<0.001
Total Dmean (Gy)	4.5 ± 0.3	4.3 ± 0.2	0.392
Total V20Gy (%)	8 ± 0.6	7 ± 0.4	0.295
Total V5Gy (%)	17.5 ± 0.7	16.6 ± 0.6	0.382
TLV (cm^3^)	2276 ± 89	3759 ± 140	<0.001

^a^=Mean ± SD.

#### OAR doses

All patients met the heart constraint dose (V25 Gy < 10%) with a maximum V25 value of 9% in the FB plan and 2% in the DIBH plan. Dose differences between DIBH and FB for the lung and the heart were analysed. Figure 3a shows the benefit obtained with DIBH by physically removing the heart from the beam trajectory. The results for the dose to the heart are shown in Table 3. Analysis of the dose differences between the CTFB and CTDIBH plans showed that the fraction of the heart volume receiving 3–40 Gy was significantly reduced by DIBH. Overall, the MHD was reduced from 2.97 ± 1.72 Gy (FB) to 1.75 ± 0.12 Gy (DIBH), resulting in a relative dose reduction of 1.22 Gy (41%) for patients receiving a total dose of 50 Gy in 2 Gy per fraction. Furthermore, as shown in Fig. 3b and c, there was a noticeable separation between the two groups when the MHD was plotted against the left and total lung volumes, with the DIBH group having a lower MHD than the FB group. In terms of the other cardiac structures, there was also a significant reduction in the dose to the LAD using the DIBH technique. On average, the Dmean of the LAD for patients receiving a total dose of 50 Gy was reduced by 12 Gy (41%). Furthermore, by using DIBH, the mean ILV of 21 patients increased by 76% (973 cm^3^*vs* 1716 cm^3^, *P* < 0.0001), and the TLV increased by 65.2% (2276 cm^3^*vs* 3759 cm^3^, *P* < 0.0001). The dosimetric analysis showed that the DIBH technique had no significant impact on the Dmean, V20 or V5 to the left or total lung when compared with the FB plan.

### Parameters used to predict patients who would benefit from DIBH in terms of ILV and TLV

**Fig. 4.**
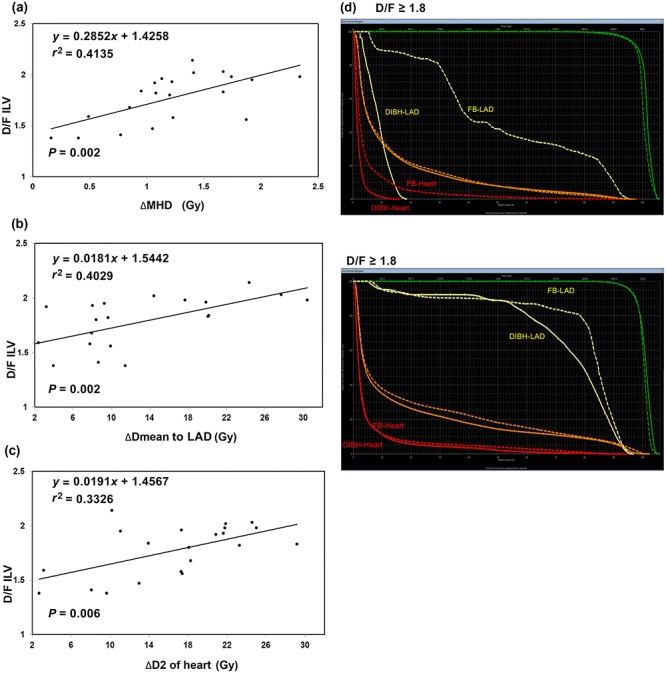
Relationship between the differences (Δ) in LAD and heart doses and the difference in ILV between FB and DIBH. The differences (Δ) in MHD (**a**), Dmean to LAD (**b**) and (**c**) and D2 of the heart (c) plotted against the difference in ILV between FB and DIBH. (**d**) The DVH comparison of IMRT treatment plans with and without DIBH gating. Left side: one patient with D/F (DIBH/FB) ILV = 1.83; right side: one patient with D/F ILV (ILV in DIBH/ILV in FB) = 1.59 (DIBH: solid line; FB: dashed line; heart: red; LAD: yellow; PTV: green; ipsilateral lung: orange). LAD = left anterior descending artery, ILV = ipsilateral lung volume, FB = free-breathing, DIBH = deep inspiration breath-hold, MHD = mean heart dose, Dmean = , D2 = near-maximum dose of 2 cc to the heart, DVH = dose–volume histogram, IMRT = intensity-modulated radiotherapy, PTV = planning target volume, DIBH-heart = DVH curve for heart based on DIBH plan.

Because lung volume was identified as the significant predictor of the dose–volume parameters observed above, we further investigated whether lung volume was a predictive parameter for identifying those patients who would benefit from DIBH. Differences in dose parameters were calculated as differences between DIBH and FB; for instance, ΔMHD (Gy) = (MHD in FB – MHD in DIBH). The differences in lung volume were calculated as lung volume during DIBH normalized to the FB values and are expressed as a percentage; D/F lung volume = [lung volume in DIBH (cm^3^)/lung volume in FB (cm^3^)]. Our data showed that the differences in lung volume were significantly correlated with the ΔMHD and ΔD2 of the heart and ΔDmean of the LAD (Fig. 4a–c). We divided the patients into two groups according to the median value of D/F ILV (1.8) and two groups of D/F TLV (1.6), the high and low groups. As shown in Fig. 4d and Tables 4 and 5, the higher D/F ILV and D/F TLV groups were significantly more likely to have larger differences in heart and LAD dose parameters between FB and DIBH plans.

**Table 4:** The doses and the differences (Δ) for LAD and heart according to the changes of ILV between FB and DIBH

	D/F ILV		*P* value
	<1.8	≥1.8	
**LAD Dmean**			
FB	30.39 ± 4.56	29.42 ± 2.78	0.944
DIBH	23.68 ± 5.2	13.5 ± 1.81	0.131
Δ mean dose	6.7 ± 1.27	16.39 ± 2.31	0.006
**MHD**			
FB	2.9 ± 0.38	3.0 ± 0.16	0.727
DIBH	2.05 ± 0.24	1.57 ± 0.15	0.057
Δ MHD	0.85 ± 0.19	1.44 ± 0.11	0.010
**D2 heart**			
FB	35.99 ± 5.42	42.2 ± 1.69	0.780
DIBH	24.79 ± 4.95	22.3 ± 2.44	0.528
ΔD2	11.19 ± 2.22	19.89 ± 1.55	0.003

**Table 5:** The doses and the differences (Δ) for LAD and heart according to the changes of TLV between FB and DIBH

	D/F ILV		*P* value
	<1.6	≥1.6	
**LAD Dmean**			
FB	31.43 ± 5.13	28.96 ± 2.61	0.829
DIBH	24.9 ± 5.83	13.65 ± 1.68	0.119
Δ mean dose	6.5 ± 1.46	15.8 ± 2.22	0.016
**MHD**			
FB	2.98 ± 0.43	2.97 ± 0.16	0.886
DIBH	2.13 ± 0.26	1.57 ± 0.11	0.033
Δ MHD	0.85 ± 0.22	1.40 ± 0.11	0.033
**D2 heart**			
FB	36.48 ± 6.24	41.5 ± 1.72	0.829
DIBH	26.29 ± 5.44	21.74 ± 2.34	0.274
ΔD2	10.18 ± 2.28	19.78 ± 1.44	0.001

^a^=Mean ± SD.

## DISCUSSION

Previous studies have shown a further reduction in cardiac exposure due to active breathing control during radiation delivery, such as DIBH [20, 24, 25]. All patients enrolled in the present study were treated with DIBH respiratory gating, and each patient had both CTFB and CTDIBH plans. It has been shown that left-sided BC is associated with eight additional deaths from ischemic heart disease per 1000 women treated, with an average MHD of 6.6 Gy [8, 26]. The lifetime risk of radiation-induced ischemic heart disease for BC patients increases linearly with an increase in the Dmean to the heart of 7.4% per Gy [8, 27]. The International Commission on Radiological Protection [28] also reported that a dose of 0.5 Gy might lead to ~1% of exposed individuals developing cardiovascular disease >10 years after exposure. As the incidence of ischemic heart disease is proportional to the MHD [7, 29], a reduction of the dose to the heart as much as possible is advisable. In our study, the MHD ranged from 1.18 to 4.43 Gy in the CTFB plan patients with left-sided BC. A comparison of all patients revealed that larger PTV volume and nodal irradiation were significantly associated with increased OAR dose. Furthermore, there was a negative correlation between the MHD and lung volume. We found that patients with a TLV ≥ 2200 ml had an MHD of 2.4 Gy; those with a lower TLV had an MHD of 3.4 Gy. These differences by TLV group were statistically significant. Czeremszynska *et al*. [30] showed correlations between PTV size and dose sparing of the heart, wherein larger lung volumes in FB had a negative effect, which agrees with our findings. Additionally, the risk of cardiac events is likely to be related to dose and irradiated volume [23]. Therefore, when evaluating breast RT planning, the mean cardiac dose is not the only factor to consider; V25 and/or V30 and V40 are also important [31]. It has been reported that a V25 Gy < 10% is likely to be associated with a <1% probability of cardiac mortality 15 years after RT [23] and that a D2 < 42 Gy for the volume constraints lowers the probability of long-term cardiac mortality to <1% [32]. In our report, an ILV ≥ 950 cm^3^ or a TLV ≥ 2200 cm^3^ was associated with lower exposure to the heart, including MHD, D2, V25 and V5 in the CTFB plans. Aside from the dose to the heart, other data suggest that the dose to the LAD poses the highest risk for developing atherosclerosis after left-sided breast RT [27, 33]. Overall, the LAD is an important structure in RT-related heart abnormalities, and the mean LAD dose observed has been substantially higher than the MHD [34]. We demonstrated a positive correlation between the MHD and the Dmean of the LAD (*r* = 0.77), where the mean Dmean ratio of LAD/MHD was ~10. There was also a negative correlation between the Dmean of the LAD and lung volume. An ILV ≥ 950 cm^3^ or a TLV ≥ 2200 cm^3^ was associated with lower dose means, and V30 and V40 to the LAD.

The DIBH technique has been shown to reduce the dose to the heart and LAD [7, 35, 36]. Our findings are consistent with studies reporting a reduction in OAR dose using the DIBH technique. In our study, a regimen using IMRT and DIBH resulted in a significantly lower MHD and Dmean of the LAD compared with FB plans. The mean dose, D2, V25, V20 and V30 of the heart significantly decreased using the DIBH technique. However, the use of DIBH had no significant impact on dose reduction of the Dmean or V5–V20 of either the ipsilateral or the total lung. This variability in dose reductions by DIBH has been previously reported in the literature [20, 35, 36]. The current study confirms that not all patients receive the same benefit from the DIBH technique and that patient outliers exist. It would be helpful to find factors to identify which patients would derive the most benefit from the DIBH technique. It has been reported that variations in chest shape can impact the cardiopulmonary dose received [19, 37]. The expansion of the lung, which shifts the heart away from the treatment field, is the main method used to decrease the heart dose through DIBH. Nevertheless, the difference in lung expansion between FB and DIBH is patient-specific. Based on the significant correlation between the lung volume and the dose to the heart and LAD, we investigated whether the change in lung volume or lung expansion during DIBH compared with FB had a significant impact on sparing the heart and LAD from irradiation. Tanguturi *et al*. [21] identified a relationship between greater inspiratory lung volumes and larger dose reductions to the cardiopulmonary structures when treating patients using the DIBH technique. In our study, differences in ILV and TLV between FB and DIBH were significantly correlated with a reduced MHD and Dmean of the LAD. Moreover, a larger D/F LV of the ipsilateral lung and of the total lung were associated with an increased difference in heart and LAD dose parameters after DIBH.

The limitations of this study were that it was a retrospective analysis with a small sample size and resource restrictions. Therefore, further investigations that include more patients in a prospective trial are needed.

## CONCLUSION

Our data demonstrated that the smaller lung volume in CTFB plans is associated with higher cardiac exposure. The data also suggested that patients who have a larger difference in ILV between DIBH and FB may obtain the greatest benefit from the DIBH technique. It suggested that lung volume (ILV and TLV) measured on a CT-simulation scan and differences between FB and DIBH could potentially be utilized to predict cardiac exposure and select which patients are likely to derive benefit from the use of DIBH.

## CONFLICT OF INTEREST

The authors declare that they have no competing interests.

## Supplementary Material

Supplementary_Figure_1_rraa003

Suppl_Table1_rraa003
