# Supplementary material for: Selection of patients with left breast cancer for IMRT with deep inspiration breath-hold technique
Source: J Radiat Res. 2020 Mar 3;61(3):431–9. doi: 10.1093/jrr/rraa003 (PMC7299258; doi:10.1093/jrr/rraa003)

**Suppl. Figure 1 Relationship between MHD, Dmean of LAD and TLV in CTFB plans for left-sided breast cancer patients**


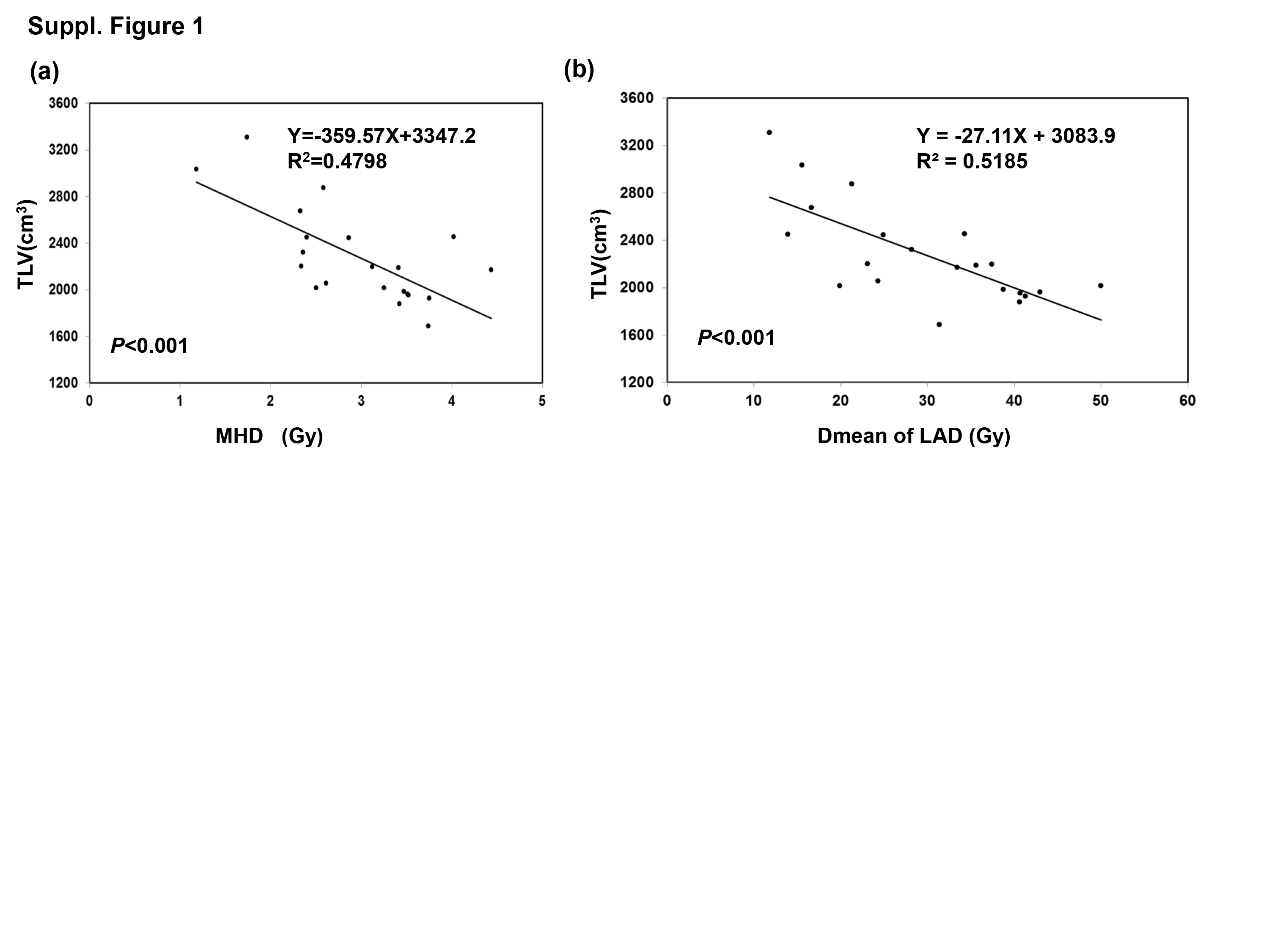


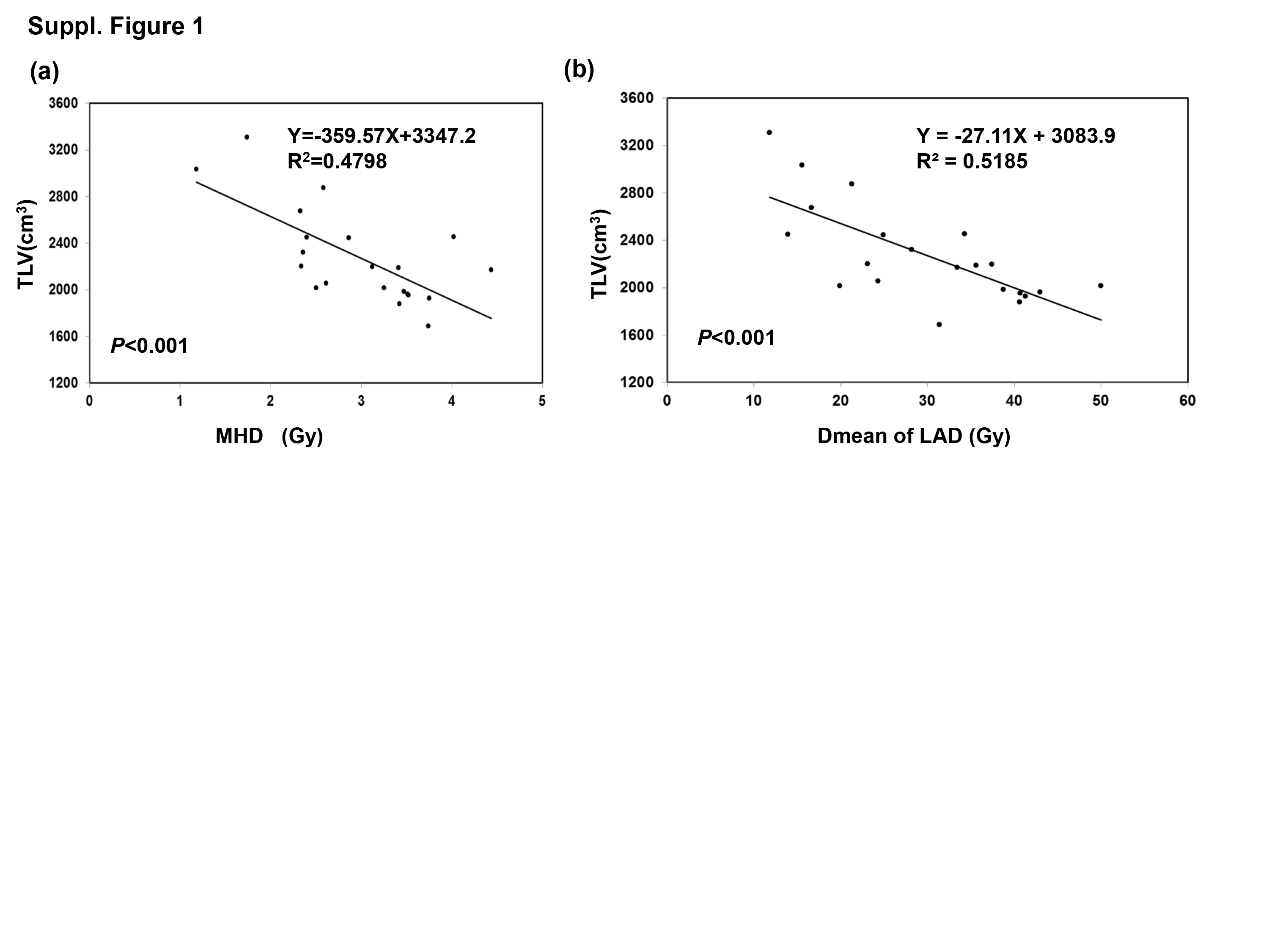

Supplement: Supplementary_Figure_1_rraa003 [file supplementary_figure_1_rraa003.docx]
