# Supplementary material for: Selection of patients with left breast cancer for IMRT with deep inspiration breath-hold technique
Source: J Radiat Res. 2020 Mar 3;61(3):431–9. doi: 10.1093/jrr/rraa003 (PMC7299258; doi:10.1093/jrr/rraa003)
Supplement: Suppl_Table1_rraa003 [file suppl_table1_rraa003.docx]

**Suppl. Table 1 Factors associated with dosimetric differences for OAR from CTFB plans**

|  | **Surgery**  **(BCS vs Mastectomy)** | **Nodal irradiation**  **( No vs Yes)** | **PTV** |
| --- | --- | --- | --- |
| **Heart** | ***P* value** | ***P* value** | ***P* value** |
| Dmean | 0.772 | 0.007* | 0.001* |
| D2 | 0.698 | 0.005* | 0.003* |
| V5Gy | 0.772 | 0.019* | 0.008* |
| V20Gy | 0.999 | 0.036* | 0.007* |
| V25Gy | 0.847 | 0.015* | 0.004* |
| **LAD** |  |  |  |
| Dmean | 0.847 | 0.036* | <0.001* |
| V30 | 0.772 | 0.12 | <0.001* |
| V40Gy | 0.961 | 0.056 | 0.002* |
| **Lung** |  |  |  |
| Left Dmean | 0.280 | 0.002* | 0.225 |
| Left V20Gy | 0.628 | 0.023* | 0.134 |
| Left V5Gy | 0.496 | 0.001* | 0.085 |
| ILV | 0.435 | 0.266 | 0.037* |
| Total Dmean | 0.327 | 0.003* | 0.336 |
| Total V20Gy | 0.496 | 0.019* | 0.195 |
| Total V5Gy | 0.237 | 0.001* | 0.360 |
| TLV | 0.496 | 0.120 | 0.028* |
